# Neuronal network information processing through heterogeneities and resonance frequency shifts

**DOI:** 10.1186/1471-2202-14-S1-P361

**Published:** 2013-07-08

**Authors:** Elizabeth Shtrahman, Michal Zochowski

**Affiliations:** 1 Department of Physics, University of Michigan, Ann Arbor, MI 48109, USA; 2 Department of Physics, University of Michigan, Ann Arbor, MI 48109, USA

## 

How can groups of neurons selectively encode different memories? We investigated a possible mechanism for the selective activation of regions of a network based on the resonance properties of individual neurons and heterogeneities in the network connectivity. In network simulations of coupled resonate and fire neurons, we incorporated the experimentally observed phenomena of resonance frequency shift based on membrane voltage changes. We aim to understand to what extent the resonance frequency shift allows for the separation of signals. We find that formation of neuronal subgroups, whether through higher connectivity strength or number of connections can lead to different activation properties from the rest of the network.

